# Photodynamic Therapy in Primary Cutaneous Skin Lymphoma—Systematic Review

**DOI:** 10.3390/jcm14092956

**Published:** 2025-04-24

**Authors:** Adam Zalewski, Witold Musiał, Alina Jankowska-Konsur

**Affiliations:** 1 Clinical Department of Oncodermatology, University Centre of General Dermatology and Oncodermatology, Wroclaw Medical University, Borowska 213, 50-556 Wrocław, Poland; alina.jankowska-konsur@umw.edu.pl; 2 Department of Physical Chemistry and Biophysics, Wroclaw Medical University, Borowska 211A, 50-556 Wrocław, Poland; witold.musial@umw.edu.pl

**Keywords:** cutaneous lymphoma, photodynamic therapy, CTCL, CBCL, lymphomatoid papulosis, PDT, 5-ALA, MAL

## Abstract

**Background/Objectives:** Primary cutaneous lymphomas (CLs) are a group of skin-limited lymphoproliferative disorders, including cutaneous T-cell (CTCLs) and B-cell lymphomas (CBCLs). Photodynamic therapy (PDT), a non-invasive, light-activated treatment, has gained attention as a skin-directed therapy for early-stage CLs due to its selectivity and favorable safety profile. This systematic review evaluates the current evidence on the clinical use of PDT in managing CLs. **Methods:** A systematic literature search was conducted in PubMed, Scopus, and Embase through 1 September 2024 following PRISMA guidelines. Search terms included “primary cutaneous skin lymphoma”, “CTCL”, “CBCL”, “mycosis fungoides”, “lymphomatoid papulosis”, and “photodynamic therapy”. After screening 1033 records, 30 studies were included. Data were extracted and categorized by lymphoma subtype and clinical outcomes. **Results:** Of the included studies, 23 focused on mycosis fungoides (MF), 5 on lymphomatoid papulosis (LyP), and 2 on CBCL. PDT demonstrated notable clinical efficacy in early-stage and localized disease, particularly MF, using methyl aminolevulinate (MAL) or 5-aminolevulinic acid (5-ALA) as photosensitizers. Adjunctive techniques like microneedling and laser-assisted delivery improved treatment outcomes. PDT was generally well tolerated, with mild, transient side effects; rare complications such as localized neuropathy were reported. **Conclusions:** PDT is a promising, non-invasive treatment for early-stage CLs, especially MF and indolent CBCL variants. While current evidence supports its safety and effectiveness, further comparative and prospective studies are needed to refine protocols, evaluate long-term efficacy, and compare different photosensitizers.

## 1. Introduction

Primary cutaneous lymphomas (CLs) are a heterogeneous group of lymphoproliferative malignancies, with lymphatic proliferation limited to the skin at the time of diagnosis. According to the World Health Organization (WHO) and the European Organization for Research and Treatment of Cancer (EORTC) classification, CLs are divided into cutaneous T/NK-cell lymphomas (CTCLs) and cutaneous B-cell lymphomas (CBCLs) [1,2]. Of them, CTCLs account for approximately 80% and CBCLs represents 20–25% of all CLs. The CL group is characterized by considerable diversity in terms of clinical presentation, prognosis, and treatment methods. In a significant proportion of cases, with only skin involvement and a good prognosis, the treatment of choice is skin-directed therapy (SDT) due to its favorable benefit-to-risk ratio. One of the emerging SDTs is photodynamic phototherapy.

Photodynamic therapy (PDT) has emerged as a novel, non-invasive treatment modality in dermatology, particularly for superficial skin cancers and other non-oncological dermatological conditions. It involves the local or systemic administration of a photosensitizer, a light-sensitive agent that accumulates in affected tissues. When exposed to light of a specific wavelength, the photosensitizer activates, triggering processes that selectively destroy the abnormal cells. These photocytotoxic reactions only occur within the pathological tissues where the photosensitizer is distributed, ensuring targeted cell destruction [3].

The efficacy of PDT in CLs remains an area of active investigation. While some studies have reported promising results, showing PDT to be a viable option for early-stage disease with minimal side effects, its role in the broader treatment landscape remains underexplored. As the current reports are scattered and heterogeneous, a systematic review of the available literature is warranted. This paper aims to evaluate the current evidence on the use of PDT in treating CLs, highlighting its potential benefits, limitations, and future directions.

### 1.1. Cutaneous Skin Lymphomas

#### 1.1.1. Cutaneous T-Cell Lymphoma

Cutaneous T-cell lymphomas (CTCLs) represent a rare group of non-Hodgkin lymphomas deriving from the T lymphocytes, with mycosis fungoides (MF) being the most prevalent entity, representing nearly 50% of cases and an overall incidence of approximately 5.6 per million persons [1,2]. The disease stages range from localized skin patches and plaques in early stages to advanced forms involving skin tumors, lymph nodes, or blood involvement [4]. In Table S1, basing on the WHO–EORTC classification criteria [1,2], the authors describe the TNM system used for MF staging.

The management of MF is highly stage-dependent. Early-stage disease (IA–IIA) generally has a favorable prognosis, with 5-year survival rates exceeding 80% for many patients and a median overall survival exceeding 20 years, comparable to the general population. It responds well to skin-directed therapies (SDTs), such as topical corticosteroids, phototherapy, and topical retinoids, to control symptoms like itching and lesions [5]. These approaches can provide significant quality-of-life improvements but do not typically result in a cure. Advanced-stage disease (IIB–IV) is associated with much lower survival rates and often requires systemic treatment such as chemotherapy, targeted biologics, and immunomodulatory therapies. Despite the emergence of new therapeutic options, data indicate that the median survival for stage IIB is approximately 5.96 years. In contrast, in more advanced cases, particularly stage IV, it decreases to 2.5–5 years in patients with extensive blood, lymph nodes, or visceral involvement [6,7,8].

#### 1.1.2. Cutaneous B-Cell Lymphomas

According to the WHO–EORTC classification, a group of cutaneous B-cell lymphomas comprises distinct subtypes: primary cutaneous marginal zone B-cell lymphoma (PCMZL), primary cutaneous follicle center lymphoma (PCFCL), and primary cutaneous diffuse large B-cell lymphoma, leg type (PCDLBCL-LT). PCMZL and PCFCL are indolent, with good prognosis and low relapse rates, while PCDLBCL-LT is more aggressive with poorer outcomes. The diagnostic process involves a combination of clinical evaluation, histopathological examination, and immunophenotyping [9]. Treatment strategies are based on the lymphoma subtype and extent of skin involvement, with localized therapies such as radiotherapy, PDT, or excision being effective for early-stage PCMZL and PCFCL. More aggressive CBCL types, like PCDLBCL-LT, often require systemic therapies, including chemotherapy and rituximab, especially in advanced stages [9,10].

### 1.2. Photodynamic Therapy

First discovered in 1904 by von Tappeiner, PDT has found a permanent position among dermatological therapeutic strategies. Descriptions of PDT for skin cancer treatment date back to the 1970s when it was used at the Roswell Park Cancer Institute. These trials ultimately resulted in the FDA approval of the procedure [11]. PDT relies on three crucial components for the procedure: a non-toxic dye called a photosensitizer (PS), low-intensity visible light, and oxygen within the target diseased tissue or cell [12]. Light activates the PS, causing it to form a triplet state. This can trigger two types of reactions—Type 1, producing ROS through substrate interaction, and Type 2, generating singlet oxygen by energy transfer to molecular oxygen. Type 2 is considered the main mechanism of the PDT method [13]. Once activated, PSs accumulate in cancer cells due to tumor-specific characteristics, allowing for selective targeting. ROS cause oxidative damage, mainly via mitochondria, leading to apoptosis [12,13,14]. PSs can be delivered topically, orally, or intravenously. A major advancement is the topical use of 5-aminolevulinic acid (5-ALA), converted to protoporphyrin IX (PPIX) in the heme biosynthetic pathway. Due to ferrochelatase limitations, PPIX accumulates, producing ROS upon light exposure. 5-ALA effectively penetrates the skin and accumulates in diseased tissue, especially in lesions with impaired stratum corneum, enhancing selectivity [15,16].

Methyl aminolevulinate (MAL), a lipophilic ester of 5-ALA, penetrates deeper and is effective for actinic keratosis and superficial BCC, often combined with red light. 5-ALA and MAL are approved for topical use in various formulations in Europe and North America [13,15,17]. In North America, a 20% formulation of 5-ALA is approved for actinic keratosis treatment using blue light [13]. Hemoporfin (HMME) is a newer PS with strong PDT activity and lower phototoxicity [15]. Research is exploring multifunctional PSs activated by stimuli like pH or enzymes. Electroporation and metal complexes (e.g., ruthenium (Ru(II)) and iridium (Ir(III))) enhance delivery and allow for real-time monitoring [18,19]. These compounds offer promising features like strong spin–orbit coupling, which facilitates better interactions with oxygen, as well as low toxicity, good biocompatibility, and the ability to monitor PDT in real time via fluorescence. Such advances point toward more effective and targeted PDT approaches, with the potential for broader clinical applications in cancer and other conditions [18]. Light source choice affects PDT efficacy. Options include lasers, broad-spectrum lamps, IPL, and LEDs [15,20]. LEDs are preferred for being affordable, non-heating, and adaptable [13,21]. The effectiveness of PDT is also determined by the wavelength of light used, as light penetration into tissue is highly dependent on tissue type and wavelength. The absorption spectrum of PPIX peaks at 410 nm (Soret band) and has additional absorption peaks at 505, 540, 580, and 635 nm [15,20,22]. Shorter wavelengths (below 600 nm) penetrate less effectively, while longer wavelengths (above 850 nm) lack sufficient energy to generate reactive oxygen species (ROS) in the target tissue [3,23].

A recent trend in PDT is the use of natural daylight, known as daylight PDT (dPDT). Daylight provides a convenient and less painful alternative to artificial light sources [24]. Though slower, it is effective and less painful for conditions like actinic keratosis [22,24,25]. Light fluence and fluence rate influence PDT results. Lower rates preserve oxygen and favor apoptosis over necrosis [26,27]. A proper light setup optimizes results and reduces discomfort [28].

## 2. Materials and Methods

**Figure 1:**
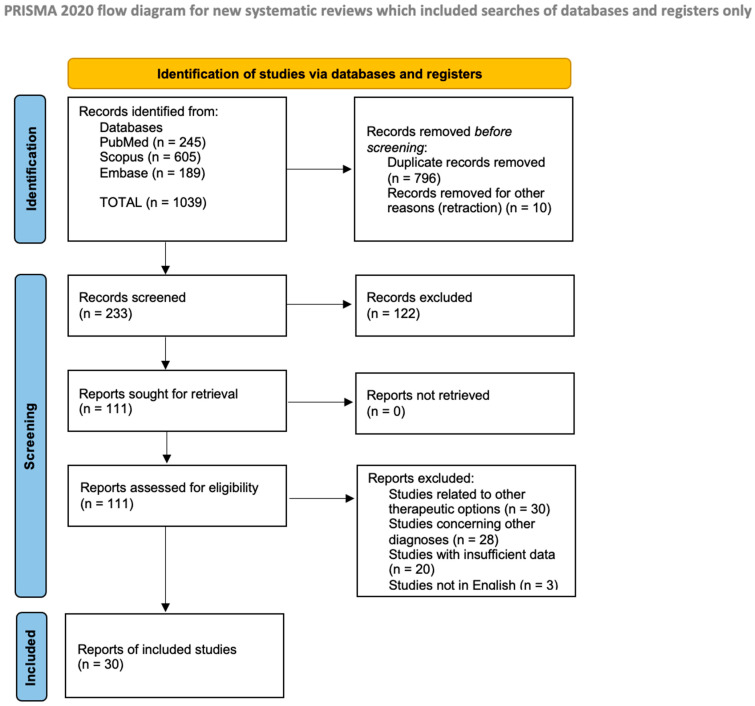
PRISMA flow diagram.

In this study, we conducted a literature review on the clinical cases concerning PDT use in CL patients, following PRISMA guidelines [29]. We searched three electronic databases—PubMed, Scopus, and Embase—on 1 September 2024 for articles published since 1990. The search utilized the following medical subject heading (MeSH) terms: “primary cutaneous skin lymphoma” OR “mycosis fungoides” OR “lymphomatoid papulosis” OR “CTCL” OR “CBCL” AND “photodynamic therapy”. Our initial search yielded 1033 articles. After removing duplicates, we were left with 227 papers. Two independent reviewers screened the studies in two phases: first based on titles and abstracts and then through full-text review. The first screening identified 105 records; however, upon a thorough review of titles and abstracts to assess eligibility—during which non-English articles and those lacking sufficient data or related to other hematological conditions were excluded—we included a total of 30 articles in our review. A PRISMA flow diagram was created to illustrate this process (Figure 1). All articles were managed using Zotero 5.0 under an AGPL license. Given the observational nature of most included studies, the certainty of evidence was rated low to moderate. A formal GRADE assessment was not performed, but limitations such as a lack of randomization or protocol heterogeneity were acknowledged. Another important limitation of this review is the relatively small sample size, with only 30 studies meeting the inclusion criteria, most of which involved limited patient numbers and case-based evidence. The absence of large-scale, randomized controlled trials (RCTs) restricts the generalizability of the findings and prevents firm conclusions regarding the comparative efficacy and long-term outcomes of photodynamic therapy in primary cutaneous lymphomas.

The review was not registered in PROSPERO or any other registry, and no separate protocol was prepared.

## 3. Results

Of all collected articles, 5 studies concerned patients with lymphomatoid papulosis, 23 records provided data about PDT therapy among patients with MF, and 2 reports related to CBCL. The analysis of the data revealed that photodynamic therapy (PDT) demonstrates significant clinical efficacy, particularly in early-stage and localized cases, with both methyl aminolevulinate (MAL) and 5-aminolevulinic acid (5-ALA) used as effective photosensitizers. The results of our research are presented in Table 1, Table 2 and Table 3.

## 4. Discussion

### 4.1. PDT in Skin Lymphomas

Photodynamic therapy (PDT) has emerged as a promising treatment option in lymphomatoid papulosis (LyP), early-stage mycosis fungoides (MF) and cutaneous B-cell lymphomas (CBCLs), including marginal-zone lymphoma (MZL)-type CBCL [58,60]. It differs in several aspects from its application in other malignancies, especially due to the hematologic origin and unique pathophysiology of the disease. Unlike epithelial tumors, CLs consist of malignant T-cells infiltrating the epidermis and superficial dermis, which may affect the uptake and distribution of topically applied photosensitizers [61]. Furthermore, due to the chronic and multifocal nature of CLs, PDT is typically used for localized, early-stage lesions, rather than as a definitive curative therapy as in actinic keratoses or basal cell carcinomas [62,63]. The precise mechanism by which PDT exerts its therapeutic effects in CLs remains not fully understood. While PDT is known to induce direct cytotoxicity through the generation of reactive oxygen species (ROS), leading to the apoptosis or necrosis of malignant cells, the relative contributions of direct tumor cell death versus immune-mediated effects are still under investigation [23]. Studies suggest that PDT may also trigger immunogenic cell death (ICD), characterized by the release of damage-associated molecular patterns (DAMPs) such as calreticulin, heat shock proteins, and high mobility group box 1 protein (HMGB1), which can enhance dendritic cell maturation and subsequent T-cell activation [61]. Immune-mediated mechanisms therefore appear to play a more prominent role in CLs than in solid tumors, with PDT-induced inflammation and cytokine release potentially enhancing antitumor immunity. The typically ill-defined and diffuse morphology of CL lesions can also complicate treatment planning, requiring individualized light delivery approaches. These differences underscore the need for disease-specific protocols and further research to elucidate the complex interplay between cytotoxic and immunomodulatory effects in PDT-treated CLs [61,63].

#### 4.1.1. PDT in Lymphomatoid Papulosis (LyP)

PDT has shown potential as a treatment for refractory LyP, particularly in cases resistant to conventional therapies. Case studies have illustrated its effectiveness across diverse patient profiles. For example, a pediatric case involving a 13-year-old boy with extensive LyP lesions, unresponsive to other therapies, achieved complete resolution in the affected area after two months of PDT in conjunction with narrowband UVB treatment, demonstrating the potential of PDT in pediatric cases due to its minimal toxicity and non-invasive nature [32].

For adult patients with treatment-resistant LyP, PDT has provided effective, localized control of lesions. A case report by Rodrigues et al. [30] described a 40-year-old woman with LyP whose persistent lesions on the abdomen were cleared after two PDT sessions, remaining in remission for 11 months post-treatment [30]. The mechanism behind PDT’s efficacy may involve immunogenic cell death, where PDT stimulates the release of DAMPs, like HMGB1 and calreticulin, which can enhance immune response and prevent recurrence [31]. However, PDT is not without risks; a rare but serious side effect was reported in a 38-year-old patient who experienced transient leg paralysis after treatment on lesions near the thoracic spine, possibly due to nerve inflammation caused by free radicals generated during PDT [33].

Collectively, these cases suggest that while PDT offers a promising option for managing LyP, especially for localized, symptomatic, and therapy-resistant lesions, careful consideration regarding treatment areas and potential side effects is required.

#### 4.1.2. PDT in Cutaneous T-Cell Lymphomas (CTCLs)

PDT has been successfully used for early-stage MF, with various studies reporting complete response rates ranging from 20–100% depending on the stage and the photosensitizer used [64,65]. The first report of beneficial ALA PDT for MF was published in 1994 [66], and since then, various studies have confirmed its efficacy. Between 1994 and 2001, several small studies and case reports demonstrated that topical 5-ALA PDT could induce both clinical and histological remission in patients with unilesional or early-stage MF [51,52,53,54,55,56,57]. For instance, Wolf et al. [52] and Stables et al. [54] noted the complete clearance of treated lesions, histologically confirmed with durable remission, while Markham et al. [57] documented the effective treatment of tumor-stage MF. Although these studies involved small numbers of patients, they offered compelling early evidence for the feasibility and therapeutic potential of PDT in CTCL and established the base for future trials. Another notable example of PDT efficacy in MF is the work by Hooper et al. [67], who observed a 67% complete response rate in patients with stage IA MF. Similarly, the use of silicon phthalocyanine (Pc 4) in PDT has been tested in various studies, including those targeting MF. Pc 4, a second-generation photosensitizer, has shown partial response rates of around 40% in stage I–II MF when used in conjunction with red light (675 nm) [60,68]. Although the results from clinical trials are promising, further studies are needed to better understand the optimal dosages, light fluence, and mechanisms underlying PDT’s effects on lymphoma cells, as well as its long-term impact on disease recurrence.

A retrospective study of Barrachin et al. [35] focused on the efficacy and safety of using MAL as a photosensitizer for PDT in patients with early-stage MF. The results showed significant clinical improvements in lesion appearance, with a high tolerance among patients. The study concluded that MAL PDT could be a viable non-invasive treatment option, reducing the need for more aggressive therapies and offering a favorable safety profile [35]. It may also be an option for treating cervical and facial lesions associated with folliculotropic MF as well as uni- or paucilesional MF [41,42,43,47,50,69]. There are also studies demonstrating effective results, supporting the application of PDT in sensitive facial areas with erosive lesions where conventional treatments might be inadequate [49]. In another study concerning PDT use in MF patients, the authors presented two case studies where patients diagnosed with follicular mucinosis—a distinct variant of MF—were treated with PDT [36]. The treatment resulted in notable clinical improvement, suggesting that PDT is effective for this less common presentation of MF. This study underscores the versatility of PDT beyond typical MF cases, indicating its potential application in treating related skin conditions [36]. In the study by Dairi et al. [37], the authors utilized an ablative fractional CO2 laser (AFL) in conjunction with PDT to treat localized MF. This approach aimed to enhance the penetration of the photosensitizer and improve treatment efficacy, particularly for resistant lesions. The combination therapy demonstrated promising results, showing both clinical and histological improvements in treated patients, indicating that an AFL can significantly improve the effectiveness of PDT for localized MF [37].

Kim et al. [38] addressed a letter concerning a specific case where a patient with MF exhibited an incomplete response to topical 5-ALA therapy. The authors emphasized the variability in patient responses to topical treatments and advocated for alternative therapies, such as PDT, to achieve better outcomes for patients who do not adequately respond to standard treatments [38]. In 2016, Han et al. [39] reported on three patients with refractory-plaque-stage MF who underwent PDT. They highlighted PDT’s potential as an effective option for patients who have not responded to traditional therapies, demonstrating its efficacy in more resistant forms of MF [39]. Moreover, results of 5-ALA use in PDT showed significant improvements in both the clinical symptoms and histological features of the disease [44,48].

The way of administering the photosensitizer was evaluated through the intradermal application of 5-ALA in PDT for treating tumor lesions in MF patients [40]. The findings revealed notable reductions in lesion size and improved appearance, indicating that the intradermal method enhances the penetration of the photosensitizer, resulting in superior clinical outcomes compared with topical applications alone [40].

A case report by Paech et al. [45] described a patient with advanced HIV and MF who achieved remission after receiving topical 5-ALA and PDT. Results suggest that PDT can be effective even in immunocompromised patients, thus broadening the applicability of this therapy in complex cases of MF [45].

Not only conventional but also daylight PDT can bring satisfactory results in treating palmoplantar lesions in MF patients [46]. The findings of a study from 2021 indicated that PDT effectively managed challenging skin lesions, providing a non-invasive treatment alternative that was well received by patients [46].

Overall, the expanding role of photodynamic therapy in managing mycosis fungoides has been illustrated, providing evidence for its efficacy across different forms and stages of the disease. The findings underscore the versatility of PDT as a non-invasive option, particularly for localized or less common presentations of MF, thereby enhancing treatment options for patients.

#### 4.1.3. PDT in Cutaneous B-Cell Lymphomas (CBCLs)

While PDT has shown more established results in treating CTCLs, its application in CBCLs, such as marginal-zone lymphoma (MZL), is relatively novel but promising. The first successful ALA PDT treatment of early-stage CBCL was reported in 2006 [59]. More recently, Toulemonde et al. [58] conducted a case series of MZL-type CBCL patients treated with PDT, yielding favorable outcomes. PDT in CBCL works similarly to CTCL by using photosensitizers like 5-ALA or MAL, which accumulate in the lymphoma cells and are activated by light to induce apoptosis. One challenge in treating CBCL with PDT is the deeper localization of atypical lymphocytes, which can make it harder for photosensitizers to penetrate the skin’s deeper layers [58]. To overcome this, some studies have incorporated microneedling before PDT, which enhances the skin’s permeability and boosts photosensitizer uptake, leading to improved therapeutic outcomes [58,70,71].

In a study of four patients with MZL-type CBCL, all were treated with MAL PDT after presenting with multiple skin lesions, making PDT a preferred option over surgery or radiotherapy [58]. The patients, aged 27 to 64, underwent skin biopsies to confirm diagnosis, and two had previously received rituximab. A dermaroller was used to enhance MAL penetration, followed by illumination using an AKTILITE device. Each patient underwent multiple PDT sessions, and the results showed varying degrees of effectiveness. Two patients achieved complete clinical and histological remission, one showed clinical improvement but had histologically persistent disease, and one had a mixed response with partial lesion resolution. No new lesions developed during treatment, and the average pain reported was moderate, with one patient discontinuing two sessions due to pain. This approach has the potential to reduce PDT dosage and side effects by allowing for lower concentrations of photosensitizers and light exposure. However, recurrence remains a concern, as PDT, like other localized treatments (e.g., corticosteroids or topical imiquimod), primarily targets visible lesions and does not prevent distant relapse [58].

#### 4.1.4. Clinical Trials

As PDT has found more and more applications in general dermatology, the following trials were conducted to underscore the potential of PDT in treating early-stage and refractory CTCL, highlighting a range of response rates depending on the stage and treatment approach.

NCT01800838: This study examined the safety and efficacy of topical silicon phthalocyanine (Pc 4) for treating IA–IIA mycosis fungoides by evaluating a protocol of dosage acceleration. All participants (n = 11) completed the trial without experiencing any serious adverse effects. The maximum tolerated dose (MTD) for photodynamic therapy (PDT) was established at 150 J/cm^2^, while for Pc 4, it was 0.1 mg/mL [72].

NCT03281811: This trial investigated the effects of ALA PDT on refractory tumors and plaques in patients with mycosis fungoides, demonstrating a moderate response rate of 36.4% (measured with PGA). Treatment was repeated every 4 weeks. Up to six cycles were performed. At week 24, patients started radiation therapy and continued it daily for 4 weeks [73,74].

NCT00103246: This study focused on the safety profile and tolerability of Pc4 administered topically before red light PDT in subjects with various non-melanoma skin neoplasms and pre-neoplastic conditions. Particularly beneficial results were observed among MF patients where 14 of 35 (40%; 95% CI: 0.26–0.56) subjects showed a clinical response, with a good treatment tolerability [75].

NCT00023790: The aim of this study was to determine the maximum tolerated dose of Pc4 with a fixed dose of light and the dose of light when administered with a fixed dose of the drug. Unfortunately, the trial was terminated due to slow patients’ accrual [76].

NCT00054171: This study was constructed to evaluate the effectiveness of PDT with short (1–2.5 h), medium (4–6 h), and long (18–24 h) applications of 20% ALA in CTCL/CBCL and early chronic leukemia with the skin involvement. Determining the maximal irradiance and corresponding exposure among multiple treatments as well as the number of sessions needed to complete treatment were one of the study’s objectives. Nevertheless, no results have yet been posted [77].

NCT02448381: This trial centered on the efficacy of synthetic hypericin, with response rates increasing with additional treatment cycles, particularly for early-stage CTCL. After three cycles of the therapy, the response rate was assessed at 49% of the study participants. Together with a low rate of AEs, the efficacy of this treatment modality may indicate that synthetic hypericin PDT may be an effective option for MF/CTCL management [78].

NCT05380635: This study investigated the safety of 0.25% hypericin topical ointment used twice a week for 8 weeks, followed by visible light radiation. It was found that 25.9% (n = 7) of patients achieved ≥50% improvement in disease severity, and 14.8% (n = 4) reached 100% improvement. Mild local reactions but no severe adverse effects were reported [79].

These trials underscore the potential of PDT in treating early-stage and refractory CTCL, highlighting a range of response rates depending on the stage and treatment approach.

#### 4.1.5. Adverse Effects Associated with PDT

While generally well tolerated, PDT can be associated with several adverse effects [80,81]. Below in Table 4, the authors summarize the potential side effects of PDT treatment.

#### 4.1.6. Treatment Limitations

Despite encouraging results from small-sample studies, no comparative data currently exist evaluating the efficacy of PDT against other well-established skin-directed therapies for early-stage cutaneous lymphomas. Standard topical treatments such as corticosteroids, chlormethine (mechlorethamine), calcineurin inhibitors (e.g., tacrolimus), imiquimod, ingenol mebutate, and localized radiotherapy including electron beam therapy have been used with varying success in early-stage MF, yet head-to-head studies comparing these modalities with PDT are lacking [1,6,9,82]. The absence of comparative trials limits the ability to position PDT in the current therapeutic algorithm and underlines the need for randomized studies to determine its effectiveness, safety profile, and long-term outcomes compared with existing topical treatments [80,83].

Moreover, PDT is an office- or hospital-based procedure. The administration of photosensitizers and the use of controlled light sources require a clinical setting equipped with an appropriate devices and trained personnel [83]. Additionally, patient monitoring during and after illumination is essential due to the possibility of side effects such as pain or erythema [33]. These requirements are in line with current dermatological guidelines, which recommend that PDT be performed in specialized healthcare facilities to ensure safety and treatment efficacy [80].

## 5. Conclusions

In conclusion, PDT represents a promising, non-invasive treatment option for both CTCLs and CBCLs, particularly in early-stage disease. While PDT for MF and MZL-type CBCL has shown encouraging results, further studies are needed to refine treatment protocols, optimize photosensitizer use, and address challenges like disease recurrence. The development of new photosensitizers, combined with innovative techniques like microneedling, could enhance the efficacy of PDT and offer an alternative or adjunctive therapy for patients with localized cutaneous lymphoma. Direct comparative studies between PDT and other skin-directed therapies are limited. As of our current knowledge, there are also no direct comparative studies evaluating the effectiveness of 5-ALA versus MAL in PDT specifically for CSLs. It would be of special importance to conduct head-to-head studies to improve clinical outcomes of therapeutic management. As clinical trials continue to explore these options, PDT may become an increasingly integral part of the therapeutic landscape for cutaneous lymphomas.

## Figures and Tables

**Table 1:** Results of studies on photodynamic therapy in lymphomatoid papulosis concerned in this review.

Study Title	Authors	Treatment	Results	Number of Patients	Follow-Up Duration
Successful treatment of lymphomatoid papulosis with photodynamic therapy [30]	Rodrigues et al.	Two sessions of methyl aminolevulinate photodynamic therapy (MAL PDT) with 1 week interval	Complete clinical clearance of lesions for 11 months post-treatment	1	11 months
Photodynamic therapy can prevent recurrence of lymphomatoid papulosis [31]	Arimatsu et al.	A single session of 20% aminolevulinic acid PDT with visible light exposure	No recurrence in the treated area for one year	1	12 months
A case of pediatric lymphomatoid papulosis treated with photodynamic therapy and narrowband ultraviolet B [32]	Snider et al.	Full-body narrowband ultraviolet B and targeted 20% aminolevulinic acid PDT—a total of three treatments in biweekly intervals	Complete resolution of nodules on the right forearm within two months	1	2 years
Leg paralysis after photodynamic therapy for lymphomatoid papulosis: A case report [33]	Genco et al.	Five sessions of methyl aminolevulinate PDT at the thoracic spine level	Transient leg paralysis was observed after treatment sessions, with no recurrence of paralysis after switching treatment	1	1 month
Refractory lymphomatoid papulosis successfully treated with IFN-α2a and photodynamic therapy [34]	Cheng et al.	Combined IFN-α2a with five sessions of photodynamic therapy	Significant reduction in lesion size and inflammation following combination treatment	1	NA

**Table 2:** Results of studies on photodynamic therapy in mycosis fungoides concerned in this review.

Study Title	Authors	Type of MF	Treatment	Results	Number of Patients	Follow-Up Duration
Efficacy and tolerance of photodynamic therapy with methyl-aminolevulinic acid in early-stage mycosis fungoides [35]	Barrachin et al.	Early-stage MF	One to seven sessions of methyl aminolevulinate photodynamic therapy (MAL PDT) with 2–4 week intervals	Significant clinical improvements were observed, with 70–90% clearance of lesions in most patients; treatment was well tolerated with mild side effects	30	1 year
Follicular mucinosis successfully treated by photodynamic therapy [36]	Zhao et al.	Follicular mucinosis	Two sessions of PDT with an unspecified photosensitizer at weekly intervals	Complete resolution of lesions in both cases, indicating the effective management of a rare MF variant with PDT	2	9–10 months
Localized mycosis fungoides treated with laser-assisted photodynamic therapy [37]	Dairi et al.	Localized MF	Four to twelve sessions of laser-assisted MAL PDT with monthly intervals	Notable reduction in lesion size and improved skin appearance; laser assistance enhanced the effectiveness of PDT, leading to positive patient outcomes—remission after treatment lasted 6–18 months	4	6–18 months
Mycosis fungoides showing incomplete response to topical 5-aminolaevulinic acid phototherapy [38]	Kim et al.	Early-stage MF	Variable sessions of topical 5-aminolevulinic acid (5-ALA) PDT	Highlighted variability in treatment responses, as the patient did not achieve significant improvement; suggests need for alternative treatments like PDT	1	NA
Observation of clinical efficacy of photodynamic therapy in 3 patients with refractory-plaque-stage mycosis fungoides [39]	Han et al.	Refractory-plaque-stage MF	Two to three sessions of ALA PDT	Significant clinical responses were noted, with 2 out of 3 patients achieving over 75% reduction in plaque size; PDT provided a viable option for treatment-resistant cases	3	8–17 months
Photodynamic therapy with intradermal application of 5-aminolevulinic acid successfully improved tumor lesions [40]	Kabata et al.	Advanced MF	Four to eight sessions of intradermal 5-ALA PDT	Marked improvement in tumor lesions observed; complete or partial responses in all patients, demonstrating the benefits of enhanced photosensitizer delivery	1	NA
Photodynamic therapy with methyl aminolevulinate for cervical and/or facial lesions [41]	Debu et al.	Folliculotropic MF	Six to ten sessions of methyl aminolevulinate PDT (MAL PDT) at biweekly intervals	Effective treatment for cervical and facial lesions, with most patients reporting significant lesion reduction; however, some limitations regarding depth of penetration were noted	3	12–19 months
Photodynamic therapy with methyl aminolevulinic acid for paucilesional mycosis fungoides [42]	Quéreux et al.	Paucilesional MF	Four to six sessions of MAL PDT with monthly intervals	Excellent response rates in patients with fewer lesions; up to 80% of patients experienced clinical improvement, validating PDT’s effectiveness in less extensive disease	12	12
Photodynamic therapy with methyl aminolevulinate as a valuable treatment option for unilesional cutaneous T-cell lymphoma [43]	Zane et al.	Unilesional MF	PDT with a 20% MAL was repeated once weekly until the complete clearing of the lesions or, in the case of partial clearing, when three successive treatments provided no further improvement	The study supports PDT as a feasible and effective treatment for unilesional MF, with 75% of patients achieving significant lesion improvement	5	12–34 months
Photodynamic therapy with topical 5-aminolevulinic acid for mycosis fungoides: clinical and histological response [44]	Edström et al.	Early-stage MF	Two to eleven sessions of topical 5-ALA PDT at biweekly intervals	Demonstrated both clinical and histological response; significant reductions in lesion thickness and inflammation scores were reported	10	4–21 months
Remission of a cutaneous mycosis fungoides after topical 5-ALA sensitization and photodynamic therapy [45]	Paech et al.	MF in HIV-positive patient	Two cycles of topical 5-ALA PDT	Complete remission was achieved in the patient, illustrating the potential of PDT in HIV-infected patients	1	12 months
Successful treatment of palmoplantar dyshidrotic lesions of mycosis fungoides [46]	Juan-Carpena et al.	Palmoplantar lesions	Six sessions of MAL PDT (conventional and daylight) at two-week intervals under regional anesthetic block	Effective management of difficult palmoplantar lesions; most patients reported significant relief from symptoms and reduction in lesions	1	1 year
The treatment of unilesional mycosis fungoides with methyl aminolevulinate-photodynamic therapy [47]	Hegyi et al.	Unilesional MF	Three sessions of MAL PDT within 5 weeks	At 16 months after the last session no infiltration of the treated lesion was observed, only slight discoloration; also, histopathological improvement was observed	1	16 months
Topical 5-aminolevulinic acid photodynamic therapy for the treatment of unilesional mycosis fungoides [48]	Díez Recio et al.	Unilesional MF	Three sessions of topical 5-ALA PDT with 585 nm C-Beam laser, monthly intervals	Successful in two cases, with significant lesion improvement and minimal side effects, supporting PDT as an effective option	2	NA
Topical methyl aminolevulinate-photodynamic therapy in erosive facial mycosis fungoides [49]	Debu et al.	Erosive facial MF	Six sessions in total of MAL PDT combined with systemic treatment	Effective in managing erosive lesions; notable improvements in both clinical appearance and patient comfort were observed	1	19
Unilesional plantar mycosis fungoides treated with topical photodynamic therapy [50]	Kaufmann et al.	Unilesional plantar MF	Eight sessions of topical ALA PDT at biweekly intervals	Demonstrated effective treatment in plantar lesions, leading to significant symptom relief and lesion reduction	1	4 years
Photodynamic therapy of non-melanoma malignant tumours of the skin using topical delta-amino levulinic acid sensitization and laser irradiation. [51]	Svanberg et al.	Early-stage MF	One to two topical ALA PDT	Two of four treated lesions cleared completely; two showed partial response	2	6–14 months
Photodynamic therapy for mycosis fungoides after topical photosensitization with 5-aminolevulinic acid. [52]	Wolf et al.	Unilesional MF	Four to five ALA PDT sessions	All three treated lesions showed complete clinical and histologic remission	2	8–14 months
Photodynamic therapy for mycosis fungoides after topical photosensitization with 5-aminolevulinic acid. [53]	Ammann and Hunziker	Unilesional MF	One ALA PDT session	Lesion showed clinical clearance post-treatment, but histologic response was not confirmed	1	NA
The treatment of cutaneous T-cell lymphoma by topical aminolaevulinic acid photodynamic therapy. [54]	Stables et al.	Unilesional MF	One ALA PDT session	Complete response with sustained remission and histological clearance confirmed at 12 months	1	12 months
Photodynamic therapy utilising topical delta-aminolevulinic acid in non-melanoma skin malignancies of the eyelid and the periocular skin [55]	Wang et al.	Early-stage MF	Three ALA PDT sessions	All 33 lesions in one patient cleared completely; excellent cosmetic results; no recurrence reported	1	17–33 months
Photodynamic therapy of cutaneous lymphoma using 5-aminolaevulinic acid topical application [56]	Orenstein et al.	Unilesional MF	One to two ALA PDT sessions	All six treated lesions achieved complete clinical remission; no relapse observed during follow-up	2	24–27 months
Topical 5-aminolevulinic acid photodynamic therapy for tumour stage mycosis fungoides [57]	Markham et al.	Tumor-stage MF	Five ALA PDT sessions	Substantial improvement with complete remission of treated lesion; histologic resolution confirmed	1	12 months

**Table 3:** Results of studies on photodynamic therapy in cutaneous b-cell lymphomas concerned in this review.

Study Title	Authors	Treatment	Results	Number of Patients	Follow-Up Duration
Photodynamic therapy for the treatment of primary cutaneous B-cell marginal zone lymphoma: A series of 4 patients [58]	Toulemonde et al.	MAL PDT was applied after microneedle abrasion every 2 or 4 weeks until six illuminations were completed	Partial and complete responses were observed; 50% of patients achieved clinical and histological remission	4	3.5–16 months
Topical photodynamic therapy for primary cutaneous B-cell lymphoma: A pilot study [59]	Mori et al.	One or two sessions of 20% ALA or its methyl ester PDT	Complete remission in all three patients, a maximum of two sessions needed	3	8–24 months

**Table 4:** Adverse effects associated with PDT use [80,81].

Adverse Effect	Description
Pain during treatment	Patients may experience burning or stinging sensations during light exposure, which can sometimes lead to treatment interruption.
Erythema and edema	Redness and swelling at the treatment site are common and typically resolve within a few days post-treatment.
Crusting and peeling	The treated area may develop crusts or peel as part of the healing process.
Pigmentary changes	Hyperpigmentation or hypopigmentation can occur, especially in individuals with darker skin types.
Infection	Although rare, secondary bacterial infections can develop at the treatment site.
Allergic reactions	Contact dermatitis or allergic reactions to the photosensitizing agent have been reported in some cases.
Ulceration	Infrequently, ulceration at the treatment site may occur, particularly if higher light doses are used.
Photosensitivity	Patients may experience increased sensitivity to light, necessitating the avoidance of direct sunlight and strong indoor lighting for a period post-treatment.

## Supplementary Materials

The following supporting information can be downloaded at: https://www.mdpi.com/article/10.3390/jcm14092956/s1, Table S1: The table provides a concise view of the TNMB system used for mycosis fungoides staging, where early stages focus on skin involvement and advanced stages involve lymph nodes, blood, and visceral organs, based on WHO–EORTC classification criteria [1,2].

## Abbreviations

The following abbreviations are used in this manuscript:

CL	cutaneous lymphoma
WHO	World Health Organization
EORTC	European Organization for Research and Treatment of Cancer
CTCL	cutaneous T/NK-cell lymphoma
CBCL	cutaneous B-cell lymphoma
SDT	skin-directed therapy
PDT	photodynamic therapy
MF	mycosis fungoides
PCMZL	primary cutaneous marginal zone B-cell lymphoma
PCFCL	primary cutaneous follicle center lymphoma
PCDLBCL-LT	primary cutaneous diffuse large B-cell lymphoma, leg type
PS	photosensitizer
1PS	singlet excited state
3PS	triplet state
ROS	reactive oxygen species
5-ALA	5-aminolevulinic acid
PPIX	protoporphyrin IX
MAL	methyl aminolevulinate
HMME	hematoporphyrin mono-methyl ether
IPL	intense pulsed light
LEDs	light-emitting diodes
dPDT	daylight PDT
MeSH	medical subject heading
LyP	lymphomatoid papulosis
DAMPs	danger-associated molecular patterns
