# Supplementary material for: Photodynamic Therapy in Primary Cutaneous Skin Lymphoma—Systematic Review
Source: J Clin Med. 2025 Apr 24;14(9):2956. doi: 10.3390/jcm14092956 (PMC12073078; doi:10.3390/jcm14092956)
Supplement: Supplementary file 1 [file jcm-14-02956-s001.zip › tableS1 2.pdf]

Stage	Tumor (T)	Node (N)	Metastasis (M)	Blood (B)	Description
IA	T1: Patches/plaques < 10% of skin<br>T1a: patches only<br>T1b: plaques only	N0: No lymph node involvement	M0: No metastasis	B0/B1: No or low Sezary cells<br>B0: <5% peripheral blood lymphocytes atypical<br>B0a: clone negative<br>B0b: clone positive<br>B1: >5% of lymphocytes atypical but <1000/μl<br>B1a: clone negative<br>B1b: clone positive	Less than 10% skin involvement, no significant blood or lymph node involvement.
IB	T2: Patches/plaques > 10% of skin<br>T2a: patches only<br>T2b: plaques only	N0: No lymph node involvement	M0: No metastasis	B0/B1: No or low Sezary cells	More than 10% of skin involved, no significant blood or lymph node involvement.
IIA	T1/T2: Patches/plaques < or > 10% of skin	N1/N2: Abnormal nodes, no lymphoma<br>N1: no histological evidence of MF<br>N1a: clone negative<br>N1b: clone positive<br>N2: early involvement with MF, aggregates of atypical cells with preservation of nodal architecture<br>N2a: clone negative<br>N2b: clone positive	M0: No metastasis	B0/B1: No or low Sezary cells	Skin lesions with abnormal lymph nodes but no lymphoma present.
IIB	T3: Tumors on the skin, lesions >1cm diameter with deep infiltration	N0-N2	M0: No metastasis	B0/B1: No or low Sezary cells	Presence of skin tumors, with or without lymph node involvement.
IIIA	T4: Erythroderma (≥ 80% skin involved)	N0-N2	M0: No metastasis	B0: No or low Sezary cells	Generalized erythroderma with or without abnormal lymph nodes.
IIIB	T4: Erythroderma	N0-N2	M0	B1	Generalized erythroderma with or without abnormal lymph nodes and blood involvent.

Stage	Tumor (T)	Node (N)	Metastasis (M)	Blood (B)	Description
IVA1	T1-T4: Any level of skin involvement	N0-N2	M0: No metastasis	B2: >1000/µl of circulating atypical lymphocytes (Sezary cells)	Extensive skin and lymph node involvement, but no metastasis. Sezary cells present in the blood.
IVA2	T1-T4: Any level of skin involvement	N3: Lymph nodes involved with effacement of normal architecture	M0: No metastasis	B0-B2	
IVB	T1-T4: Any level of skin involvement	N0-N3: Any level of node involvement	M1: Metastasis present	B0-B2	Visceral (organ) involvement, regardless of skin or lymph node involvement.

Table S1. The table provides a concise view of the TNMB system used for Mycosis Fungoides staging, where early stages focus on skin involvement, and advanced stages involve lymph nodes, blood, and visceral organs, based on WHO-EORTC classification criteria [1,2].
